# Metaphor production in the bilingual acquisition of English and Polish

**DOI:** 10.3389/fpsyg.2023.1162486

**Published:** 2023-08-01

**Authors:** Dorota Gaskins, Gabriella Rundblad

**Affiliations:** Education, Communication and Society, King’s College London, London, United Kingdom

**Keywords:** metaphor, production, bilingual, acquisition, English, Polish

## Abstract

Metaphor acquisition research has focused mostly on metaphor comprehension in monolingual children. Ours is the first study to examine metaphor production in young bilinguals. A quantitative method was employed whereby sixty-two children aged three to six, with English and Polish, were tested on their ability to produce primary (e.g., a *long day*) and perceptual resemblance metaphors (e.g., You’re my *sunshine*) in response to elicitation tasks. A univariate ANOVA revealed that the main factors to affect the production of conventional metaphors in bilingual children are their chronological age and their verbal skills in both English and Polish. No significant effect was found for nonverbal IQ, metaphor type, or testing language. These results are discussed in the context of both Conceptual Metaphor Theory, which has been concerned with the study of primary (and other conceptual) metaphors, and Structure Mapping Theory, which has focused on the use of perceptual resemblance metaphors. Usage-Based Theory is brought in to explain lexical effects in metaphor production.

## 1. Introduction

Metaphor use is not restricted to poetry. Metaphors govern the way we think and speak, from the most intellectual endeavors to the most mundane (Lakoff and Johnson, 2008). They bring stories to life, make complex arguments easier to follow, and weave through everyday language.

The potential of metaphor to entertain, clarify and illustrate lies in the capacity of words to take on several meanings, making polysemy their key ingredient. When calling a child *treasure*, for example, we express how precious they are to us, as the word *treasure* can refer to a valued possession as well as a loved person. Likewise, when describing someone as a *warm* person, we commend their ability to relate to other people, as *warm* conveys both a high temperature and an emotional availability. Most metaphors sound familiar as they have been conventionalized through frequent use; novel metaphors, which have not been previously registered in the given speech community, are exceptionally infrequent in everyday language (Kaal and Dorst, 2012), but more common in fiction writing (Dorst, 2015).

Research in metaphor acquisition has enjoyed considerable interest; inspite of this, the vast majority of studies have focused on metaphor comprehension (e.g., Almohammadi et al., n.d.; Pearson, 1990; Özçalişkan, 2002, 2005, 2007; Rundblad and Annaz, 2010; Pouscoulous, 2011, 2014; Stites and Özçalişkan, 2012; Van Herwegen et al., 2013; DiPaola et al., 2019; Lecce et al., 2019; Pouscoulous and Tomasello, 2019; Del Sette et al., 2020; Pastor et al., 2020), with much less work dedicated to metaphor production (but see, e.g., Gottfried, 1997). However, studying metaphor production is equally important as it can help us to determine when children start to use their first metaphors for negotiating their growing understanding of the world, and to establish what underlying factors help them to develop this ability. Children are unlikely to be just passive recipients of knowledge; they are bound to negotiate their understanding at school (Ritchie, 1994) and employ metaphors in the process (Carico, 2001).

As metaphor studies have focused mainly on monolingual children, those looking at their bilingual peers have remained exceptionally infrequent (Bountrogianni, 1988; Johnson, 1989; Antoniou et al., 2020). However, studying bilingual development can greatly contribute to the advancement of metaphor theory. As young bilinguals have one set of cognitive skills, but two asymmetrical pools of lexical resources in each language (Bedore et al., 2012; Hoff et al., 2012; Thordardottir, 2014), bilingual development can help us to disentangle the impact of cognitive versus lexical skills on their metaphor acquisition. Their unique type of linguistic constellation can reveal which metaphors are a product of domain-general cognitive processes developed similarly in both languages, and which are acquired asymmetrically just like any other type of lexical items.

Research has shown that metaphor use plays a crucial role in learning (Cameron, 2003; Nacey, 2013), helping monolingual children to engage with subjects such as maths (English, 1997) and science (Taylor and Dewsbury, 2018; Deignan and Semino, 2020). Considering that bilingualism is becoming more of a norm than an exception (Li, 2007; Eberhard et al., 2021), it is crucial that we also understand the extent of metaphor use in children from migrant families and other types of multilingual households. Bilingualism continues to be associated with language deficiency; bilingual children tend to be placed in low ability sets in schools (Mehmedbegović, 2009) and incorrectly referred to speech and language therapy (Stow and Dodd, 2005; Pert and Bradley, 2018). In the area of metaphor use, the needs of young bilinguals are also seen as comparable to children with Asperger’s, autism, and dyslexia (Bielenda-Mazur and Orłowska-Popek, 2020), which may add to such incorrect referrals. It is crucial to explain what aspects of metaphor acquisition are important in bilingual development so we can recommend relevant educational interventions, reduce inequality, and unlock the cognitive potential of school starters from migrant backgrounds.

Our article contributes to the current state of knowledge about metaphor acquisition by testing children’s metaphor production through a newly developed elicitation task. The focus of our study is a Polish community residing in the United Kingdom, whose three-to six-year-old children speak Polish and English on a daily basis. We test children’s ability to produce different metaphor types in response to auditory and visual stimuli and discuss our findings in light of current theoretical accounts.

### 1.1. Different metaphor types

The concept of metaphor is not monolithic; there is a broad range of metaphoric expressions in the language we speak, analyzed under different names and from different theoretical perspectives. In this study, we will attempt to synthesize the research literature on the topic, by dividing metaphors into two broad categories, perceptual and primary conceptual metaphors.

The first category is that rooted in a perceptual similarity between two distinct entities, called resemblance, or analogical, metaphors by Grady (2005), and perceptual resemblance metaphors, more recently, by Littlemore (2019). This group includes, for example, the most commonly studied nominal A-to-B metaphors, where a target concept is explained and understood through a mapping with the source concept. To describe a person, for example, one may rely on the salient qualities of a specific animal to illustrate the person’s physical attributes (e.g., She’s a *giraffe*, i.e., very tall) or of a supernatural being to illustrate the person’s relational properties (e.g., She’s an *angel*, i.e., kind, and loving; see, e.g., Lecce et al., 2019; Del Sette et al., 2020). While acknowledging clear differences in the two types of perceptual metaphors, this article treats both types as one group, capitalizing on the observation that the mappings they reflect cannot be found anywhere else in the given language; each of them in unique to the given metaphor. Proponents of Structure Mapping Theory (SMT) argue that the processing of such perceptual resemblance (henceforth: perceptual) metaphors depends on establishing a structural alignment between two notions (e.g., child versus a non-human creature) and projecting inferences via skills of analogy (Gentner and Markman, 1997; Gentner et al., 2001).

The second category is that of conceptual metaphors, which systematically link not just two single concepts but two domains, one of which is concrete and the other abstract. For example, when trying to understand or explain the abstract domain of affection or emotional availability, one might reach to the concrete domain of warmth caused by a physical proximity. According to Conceptual Metaphor Theory (CMT), links between the two domains are represented in the human mind via metaphorical mappings (e.g., AFFECTION IS WARMTH) which support the processing of their linguistic manifestations (e.g., a *warm* person, or a *cold* look; Gibbs, 1999). Within the category of conceptual metaphors, this article is concerned only with primary metaphors. As they are theorized to develop early through embodiment (Grady, 2005), they should be available to very young children. Also, in theory, primary metaphorical mappings are fairly universal irrespective of the language one speaks (Lakoff and Turner, 1989; Lakoff and Johnson, 2008) so they should manifest in similar ways across languages.

Based on work with adult speakers, current models of metaphor processing make a distinction between conventional and novel metaphors of both kinds. Studies in perceptual metaphor comprehension have shown that novel perceptual metaphors are processed as a structural alignment between the source and the target, but as repeated comparisons are made between the same two entities (e.g., *treasure*, i.e., a loved person versus *treasure*, i.e., a box filled with coins), the metaphorical meaning (i.e., a loved person) gradually comes to be associated with the base form (i.e., *treasure*). Online processing is thus required when encountering novel perceptual metaphors while any encounter with conventional perceptual metaphors relies on accessing and retrieving the lexically stored product of past comparisons (i.e., words; Gentner et al., 2001). However, when it comes to conceptual metaphors, this line is much less clear; some see each encounter with any conceptual metaphor, whether novel or conventional, as an activation of primary mapping which has emerged prior to language use (Grady, 1999; Mandler, 1999; Lakoff and Johnson, 2008), and so a matter of processing. Others argue that conventional primary metaphors are retrieved and understood in the same manner as any other lexicalized expressions (e.g., Murphy, 1996; Keysar et al., 2000; Glucksberg, 2001; Jackendoff, 2002; McGlone, 2007), and only those metaphors which result from an hoc pragmatic process of constructing novel meanings should count as real instances of metaphor use (e.g., Recanati, 2004; Wilson and Sperber, 2012).

Conventional primary metaphoric expressions seem to occupy a strategic space in this debate. If their use is driven primarily by the emergence of the underlying mappings, the role of linguistic exposure should be minimal for their acquisition. But if, like perceptual metaphors, they are sensitive to the quantity of linguistic exposure, this would suggest that their production at the moment of the communicative exchange is supported by dense lexical networks in which they are embedded and that the role of underlying mappings in their acquisition is secondary. Our study will thus focus on conventional metaphors and test the role of bilingual children’s asymmetrical lexical skills and other developmental factors in metaphor production. Due to its focus on conventional metaphors, our study will reach beyond the traditional metaphor theories such as CMT and SMT and bring in Usage-Based Theory (UBT) to verify the claims of whether the acquisition and subsequent production of conventional metaphors may resemble that of any other lexical items (e.g., Murphy, 1996; Keysar et al., 2000; Glucksberg, 2001; Jackendoff, 2002; McGlone, 2007). Lexical effects in metaphor use will be explained in terms of children’s own linguistic resources, a direct consequence of their language exposure (Barlow and Kemmer, 2000; Tomasello, 2001; Bybee, 2010; Schmid, 2020).

### 1.2. Metaphor acquisition in monolingual children

Perceptual and primary metaphors are theorized to follow very different patterns in acquisition. Perceptual metaphors are, at least in theory, developed through linguistic exposure, with priority given to those based around physical properties (e.g., Sarah is a *giraffe*); relational metaphors (e.g., Sarah is an *angel*) are acquired later (Winner, 1997; Lecce et al., 2019) despite being more common in adult use (Winner, 1997). Earlier comprehension studies showed that before the age of eight, children interpret perceptual metaphors literally, that an acquired sensitivity toward contextual information developed at a later age, along with more sophisticated lexical and pragmatic skills, lead older children to activate the world knowledge necessary to recover a meaning that might differ from the literal one (e.g., Vosniadou, 1987; Winner, 1997; Levorato and Cacciari, 2002). However, more recent studies based on both highly controlled and child-friendly experimental paradigms consistent with young children’s skills and world knowledge have been able to capture emerging comprehension of novel perceptual metaphors in children as young as three, which improves alongside the developing skills of analogical perception and alternative naming (DiPaola et al., 2019; Pouscoulous and Tomasello, 2019) as well as children’s developing lexical skills (Van Herwegen et al., 2013). As each perceptual metaphor is a unique conceptual mapping between two domains, children are expected to acquire them gradually in a piecemeal fashion in response to linguistic input and in line with their developing cognitive skills.

By strong contrast, the primary mappings of conceptual metaphors are, at least in theory, developed prelinguistically by observing correlations of experience (Grady, 2005). For example, children construct a primary mapping between affection and warmth in infancy by being held closely to their parents’ skin; once such a mapping is established, it facilitates children’s processing of linguistic metaphors from the top down (Grady, 1999). In theory, this should mean that once the child has developed an underlying mapping and has encountered their first corresponding linguistic expression (e.g., a *warm* person), they should be able to produce any linguistic expressions associated with the same mapping (e.g., a *cold* look) whether or not they have encountered them in child-directed speech, and whether they are conventional or novel.

The acquisition of primary metaphors in children, however, remains largely understudied, with only a handful of exceptions. Experimental data show, for example, that three-year-old Arabic-speaking children have an ability to comprehend both conventional and novel primary metaphors to a similar extent, and that this ability reaches near adult-like levels of comprehension by the age of four, which contrasts with their poor ability to understand perceptual metaphors at a comparable age (Almohammadi et al., n.d.). Experimental data from the monolingual acquisition of Turkish complement these findings: Özçalişkan (2002, 2005) and Stites and Özçalişkan (2012) show that the conventional and novel metaphor examples used in their studies can be understood by children as young as four, as long as they are presented in contextually supported situations, with children using verbal reasoning to explain their choice in experimental tasks by the age of five. By showing that different linguistic instantiations of the same mapping follow the same developmental schedule, Özçalişkan (2005) concludes that metaphor comprehension is a domain-general capacity; once a child has understood a given mapping, they can extend it to different target domains and to different linguistic instantiations of the same metaphorical mapping.

Experimental studies of metaphor production in very young children are extremely infrequent as metaphors are difficult to elicit through experimental design, and young children are notoriously difficult to work with using experimental paradigms. A notable exception is that of Gottfried (1997) who showed that monolingual children as young as three can produce both conventional and novel perceptual metaphors when the stimuli prime the recognition of metaphoric similarity and the word production. This is also confirmed by naturalistic research: data from a two-year-old child, recorded on a dense sampling schedule between her second and third birthday, reveal high numbers of conventional primary metaphors already at the age of two, but only a few instances of conventional perceptual metaphors (Gaskins et al., 2023). Overall, these studies point towards two main conclusions: children aged three and above should respond well to metaphor elicitation tasks, and they should perform better on primary than perceptual metaphors.

### 1.3. Metaphor acquisition in bilingual children

Studies in the emergence of metaphor use in childhood bilingualism have been scarce and focused on older bilingual children, those aged 7–12 (Johnson, 1989), 8–11 (Bountrogianni, 1988), or 12 and above (Antoniou et al., 2020); they have demonstrated that at later stages of development, metaphor comprehension in bilingual children is on a par with that in their monolingual peers. These studies, however, arose in the context of research showing that the ability to comprehend and use metaphors requires a long developmental time span and appears late in the childhood (e.g., Levorato and Cacciari, 2002). Given that monolingual children can understand metaphors much earlier than previously expected (e.g., Özçalişkan, 2007; Pouscoulous and Tomasello, 2019), we anticipate that an early onset of metaphor use should also be observed in bilingual children. With this in mind, we complement the current metaphor research with a study in metaphor production in bilingual children aged three to six, asking two main research questions.

1. What underlying factors (i.e., chronological age, non-verbal IQ, or verbal ability) and external factors (i.e., metaphor type, or testing language) predict metaphor production in young bilinguals?
2. Which metaphors do children perform well on at a certain age, and is this pattern the same both for Polish and English?

In light of previous studies, we expect that children’s performance in the metaphor elicitation task can be predicted equally well by their chronological age, non-verbal reasoning, as well as verbal skills, as all these factors tend to be highly correlated (Alghamdi et al., 2021). Children should be able to produce significantly more primary conceptual than perceptual metaphors as the former are supported through embodiment and potentially also child-directed speech, while the latter are acquired solely from child-directed speech. In light of this, we would also expect the overall developmental trajectory for primary conceptual metaphors to follow a significantly steeper increase over time than that for perceptual metaphors. We expect that children will use more metaphors in one of their tested languages (the stronger one), but that this trend might be disguised by between-language similarities in children’s use of primary conceptual metaphors, which are embodiment-driven. Meanwhile, we expect clearer between-language differences in the children’s use of perceptual metaphors, as they are theorized to develop from input, which tends to be uneven in bilingual development. Considering their embodied nature, we also believe that with time, primary conceptual metaphors in English and in Polish should develop at a fairy similar rate, but perceptual metaphors may develop faster in one language than in the other. Since the metaphors chosen for our study are direct translations between English and Polish, we also expect that language skills in the tested language will support metaphor use in both languages but less for primary conceptual than perceptual metaphors.

Last but not least, we believe that the qualitative analysis of individual primary conceptual metaphors will capture their comparable use across children from the same age group. Meanwhile, more differences are expected in the same children’s use of selected perceptual metaphors.

## 2. Materials and methods

### 2.1. Participants

This project focused on the Polish community in the United Kingdom. Poland is the second most common non-United Kingdom country of birth (Migration Observatory (MO), 2021), with an estimated 2,110,270 speakers of Polish living in England and Wales alone, according to data from the latest Census (2021). In English schools, over a million pupils speak language other than English at home, of these 53,915 (0.7%) speak Polish (NALDIC, 2012).

Participant recruitment commenced upon the receipt of ethical clearance (LRS/DP-20/21-24,540) from King’s Research Office Committee. The data were collected at the end of the pandemic after families had spent close to 2 years working largely from home, and at a point when some children from exclusively Polish-speaking families would have had little contact with their English-speaking peers. Initially, 72 bilingual children from large urban areas of the Midlands and the southeast of England volunteered to take part in the study. The inclusion criteria required that they (a) be bilingual speakers of Polish and English aged three to seven, (b) have no history of language disorders, or vision and hearing problems, (c) be able to demonstrate a sustained interest in our materials, and (d) display typical cognitive and linguistic development. Upon data examination, only 62 children were deemed eligible to take part, between 14 and 17 per each age group (Table 1). All the children had been exposed to Polish and English from birth. An effort was made to ensure an even spread of ages within each age group. If the recruited child was close in age to another whose age was already represented in the existing sample, they were tested at a later stage of their development.

**Table 1:** Children’s characteristics.

Age	Number of participants	Mean age (in months)	Age range (in months)	Gender
3–4	17	40.88	37–45	11 female; 6 male
4–5	15	54.5	48–59	7 female; 9 male
5–6	14	65.33	60–71	10 female; 4 male
6–7	16	78.93	72–83	8 female; 8 male

The first two sets of criteria were checked through the Questionnaire for Caregivers of Bilingual Children (PABIQ) developed by Haman et al. (2017), and Children’s Communicative Checklist (Bishop, 2003). Based on the information obtained, five children were excluded either because they did not speak both English and Polish on a daily basis, or as their age made them ineligible to take part. Three children were further excluded as they classified as late talkers or atypical developers. To assess the third set of criteria, children heard a simple story about a boy whose favorite book was missing, and they were asked four questions about it (e.g., *Where did he find the book*?). Two children who could not answer the minimum of two out of four questions were excluded on the understanding that they would not be able to engage with the long metaphor elicitation task.

To assess the last set of criteria, children completed a series of standardized cognitive and linguistic development tests (Table 2). Raven’s Colored Progressive Matrices (RCPM; Raven and Raven, 2003) assessed their non-verbal reasoning, the British Picture Vocabulary Scale (BPVS; Dunn et al., 1997) and the British Expressive Vocabulary Test (EVT-3; Williams, 2019) measured their receptive and productive vocabulary scores in English, and the Polish Language Development Test (PTRJ; Haman and Fronczyk, 2012) measured their receptive (PTRJ-R) and productive (PTRJ-P) vocabulary scores in Polish. As RCPM are standardized only for the ages of 5;0 and above, the scores obtained from the youngest participants were examined in light of other studies that plotted typical development of non-verbal reasoning in children aged 3;0 and above (e.g., Asano et al., 2023). Only raw scores were subsequently used in the calculations. The English and Polish vocabulary tests are different in several ways. The former are designed for those aged two and above; they include 168 and 190 test items for comprehension and production respectively, and their norms are presented in centiles (on the 0–100 scale). The latter are designed for those aged four and above; they include 28 and 25 test items for comprehension and production respectively, and their norms are presented in stanines (on the 1–9 scale).

**Table 2:** Children’s raw scores in the standardized tests.

Age	Statistics	RCPM	BPVS	EVT-3	PTRJ-R	PTRJ-P
3–4	Mean	14.35	44.11	48.05	8.52	2.64
	SD	2.66	13.47	15.25	3.44	2.71
	Min	12	21	11	3	1
	Max	17	71	69	16	11
4–5	Mean	17.06	67.50	67.00	10.53	4.46
	SD	3.57	11.58	13.92	3.27	3.60
	Min	12	45	43	5	1
	Max	26	88	86	16	12
5–6	Mean	22.71	78.60	79.93	14.20	5.73
	SD	3.95	16.54	20.45	5.60	4.51
	Min	16	46	44	5	2
	Max	29	97	105	25	17
6–7	Mean	28.81	93.37	94.87	15.31	8.62
	SD	3.67	16.43	21.99	4.01	4.33
	Min	21	55	39	9	3
	Max	34	114	127	22	19

There was an expectation that young bilinguals might display asymmetrical lexical scores in their two languages (e.g., Hoff et al., 2012). To ensure they were typically developing, we included children only if they fulfilled one of the two criteria: they either performed no lower than one standard deviation below the norm on their comprehension in English, or no lower than the fourth stanine on their comprehension in Polish. As developmental disorders do not tend to be associated with high lexical scores (Sheng, 2018), those who performed above the norm were included in the study. In addition, as young bilinguals tend to have lower scores than the published monolingual norms in their word production tasks (Haman et al., 2017), we used more generous inclusion criteria for lexical production scores. Children were only excluded from the study if they had scores below two standard deviations on their production tasks in English and, at the same time, their scores fell below the second stanine in Polish. All the children tested matched our criteria. While Polish norms for children aged three to four could not be established due to the lack of normative data for this age group, all children from this age group, except one, performed within the norm in English, for which we do have norms, and the one who was dominant in Polish performed above the norm even when compared to monolingual children aged four.

Lexical production tests additionally confirmed that 52 children were dominant in English as their score put them on a higher centile in English than in Polish; four children were balanced bilinguals as their English centile score fell within the range of the Polish stanine score; and six children were dominant in Polish as their Polish stanine score was higher than their English centile equivalent. This information was also verified through reference to parental perceptions of children’s dominance supplied during the initial online interviews.

A control group of eight participants aged 18–46, four speakers of Polish and four of English, was also recruited to validate the metaphor production task and to ensure that completing it was feasible for the target group.

### 2.2. Metaphors tested in the study

Twenty metaphors were used in the study, of which ten were conventional primary metaphors (Table 3) and ten conventional perceptual metaphors (Table 4). Before the final set of metaphors was selected, 47 primary and 46 perceptual metaphors were identified in the corpora of naturalistic interactions between children and their primary caregivers stored on the CHILDES TalkBank: English examples were taken from the MPI-EVA-Manchester corpora (e.g., Lieven et al., 2009); Polish examples were taken from the Szuman corpora (e.g., Smoczyńska, 1985). The primary metaphors were built around the mappings reported in child metaphor literature (Grady, 2005; Olofson et al., 2014; Gaskins et al., 2023). The perceptual metaphors were either A-to-B nominal or verbal metaphors built around the notion of physical similarity or relational similarity (Winner, 1997; Gaskins et al., 2023), and a similarity perceived by means of vision rather than other senses, as these types of metaphors are amongst the earliest metaphors understood by children (Gentner, 1988). In selecting metaphors appropriate for the target group, the following procedure was implemented:

1. To ensure that the task was achievable to young participants, only metaphors encoded in single words (e.g., *treasure*) were selected, rather than those found in longer stretches of speech (e.g., *storm in a teacup*).
2. To ensure that the test worked in exactly the same way in Polish and English, only metaphors with translation equivalents in the other language were used (e.g., *treasure* = skarb, i.e., a person who is very dear to us).
3. To ensure that salient context could be built into the experiment to prime the retrieval of metaphoric expressions, only metaphors encoded in words children tend to be familiar with were included (e.g., treasure often features in pirate stories).

**Table 3:** Primary metaphors used in the elicitation task and their mean familiarity ratings.

Metaphor mapping	English metaphor	Mean rating	Polish metaphor	Mean rating
Time is space	*Her day was very **long**.*	5.39	*Jej dzień był bardzo **długi**.*	5.43
Linear scales are paths	*Number 4 is **after** number 3.*	5.16	*Numer 4 jest **za** numerem 3.*	5.13
Obeying is listening	*Because you never **listen**.*	5.20	*Bo się nigdy nie **słuchasz**.*	5.53
Time is motion	*We will make a snowman when winter **comes**.*	5.44	*Zrobimy bałwana jak zima **przyjdzie**.*	5.70
Pleasure is sweet	*Thank you. You’re very **sweet**.*	5.53	*Dziękuję. Jesteś bardzo **słodki**.*	5.73
More is up	*The price was too **high**.*	5.53	*Cena była za **wysoka**.*	5.50
Control is up	*I have it all **under** control.*	5.70	*Mam to wszystko **pod** kontrolą.*	5.10
Action is motion	*How is it **going**?*	5.67	*Jak ci tam **idzie**?*	5.70
Similarity is closeness	*We are very **close**.*	5.57	*Jesteśmy ze sobą bardzo **blisko**.*	5.33
Actions are objects	*You made a promise and it’s a promise you can’t **break**.*	5.73	*Dałaś słowo to tego słowa nie możesz **złamać**.*	5.76

**Table 4:** Perceptual metaphors used in the elicitation task and their mean familiarity ratings.

Metaphor type	English expressions	Mean rating	Polish expressions	Mean rating
Nominal	*My family is the greatest **treasure**.*	5.73	*Moja rodzina to największy **skarb**.*	5.56
	*You are my **sunshine**.*	5.13	*Ty jesteś moim **słoneczkiem**.*	5.27
	*You’re such a busy **bee**.*	5.10	*Ale z ciebie pracowita **pszczółka**.*	5.30
	*You are a real **star**.*	5.66	*Prawdziwa z ciebie **gwiazda**!*	5.53
	*You’re such an early **bird**.*	5.10	*Ale z ciebie ranny **ptaszek**.*	5.40
	*Lion is the **king**.*	5.26	*Lew to **król**.*	5.46
	*You’re such a **pig**.*	5.66	*Ale z ciebie **świnka**.*	5.16
Verbal	*Or she won’t be able to **catch** it.*	5.77	*Żeby zdążyła autobus **złapać**.*	5.43
	*My stomach is about to **burst**.*	5.60	*Aż brzuch mi **pęka**.*	5.53
	*You can just **drop in**.*	5.56	*Możecie do nas **wpaść**.*	5.76

To select the final 20 metaphors, a survey including the 47 primary and 46 perceptual metaphors that followed criteria 1–3 was compiled. The set was then incorporated into a survey and rated for familiarity on a six-point Likert scale, where 0 meant I do not know this word, and 6 meant I know it very well. The respondents were 96 adult native speakers of Polish and 95 adult native speakers of English recruited through social media. Two blank copies of the returned survey were excluded from the analysis. The mean average for the metaphor ratings drawn from the remaining submissions ranged on the scale from 3.53 to 5.90, with a positive distribution skew. The metaphors selected for the elicitation task came from a pool of examples whose mean familiarity rating fell within the range of 5.10–5.77, and whose mode was 6. A Mann–Whitney test carried out on the two metaphor types (primary, perceptual) in the two languages (English and Polish) showed that the selected primary metaphors were as familiar as the perceptual metaphors (*M = 5.43, SD = 1.14831* vs. *M = 5.43, SD = 0.83095*) with any differences between primary and perceptual metaphors insignificant for Polish (*p = 0.182*) or for English (*p = 0.734*).

The metaphors were then embedded in stories based on situations familiar to children, for example playing in the sand, cooking with parents, and having a family meal. The 40 stories were between 21 and 45 syllables long (*M = 30.17, SD = 5.90*). A univariate ANOVA confirmed there was no significant difference in mean syllable length between the stories based on primary and perceptual metaphors (*F_(1, 36)_ = 0.037, p = 0.848*). However, there was a significant difference between the stories based on English and Polish metaphors (*F_(1, 36)_ = 5.226, p = 0.028*), which could be attributed to the fact that English language is more analytic while Polish is more synthetic.

### 2.3. The metaphor production task

The metaphor task consisted of the total of 84 PowerPoint slides illustrated with hand-drawn black and white pictures and accompanied with narration recorded by male native speakers of Polish and English. The presentation started with two slides used as a warm-up task: the children were shown one picture at a time (e.g., that of a fridge) and they were asked to complete the sentence they heard with one word (e.g., *We have plenty of food in our* …) in the same manner as they would in the metaphor elicitation task. After the warm-up, there we also 80 slides eliciting metaphor production, and two dummy slides (explained further below; Table 5).

**Table 5:** The sequencing of all four picture blocks, from left to right.

1A	1B	1C	dummy	2A	2B	2C	1D
3A	3B	3C	2D	4A	4B	4C	3D
5A	5B	5C	4D	6A	6B	6C	5D
7A	7B	7C	6D	8A	8B	8C	7D
9A	9B	9C	8D	10A	10B	10C	9D
10D							

**Figure 1:**
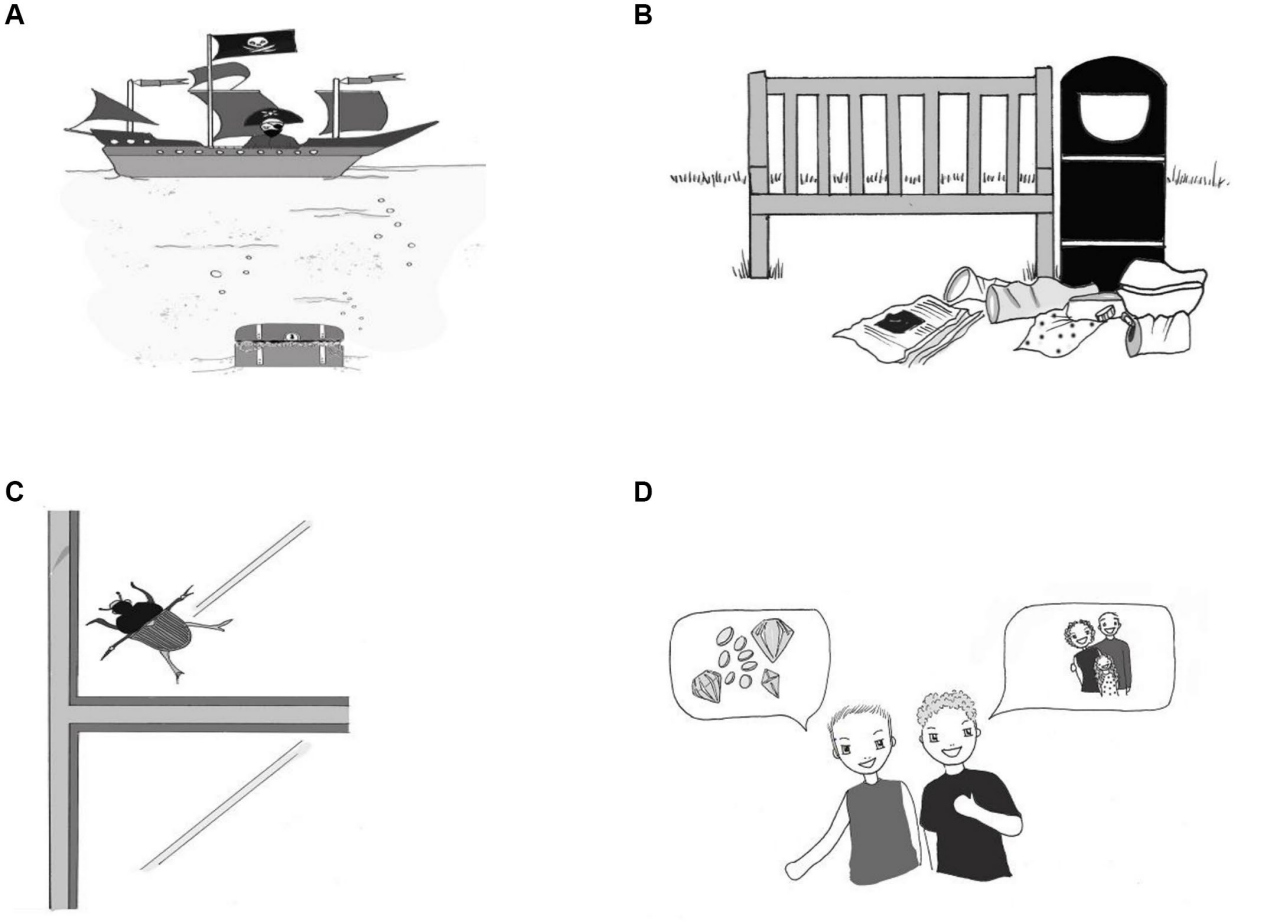
**(A)** Treasure (concrete sense); **(B)** rubbish; **(C)** beetle; **(D)** treasure (abstract sense).

After the warm-up, the children were exposed to 20 four-picture blocks. In each block, the children first saw three pictures showing concrete objects, of which one illustrated a polyseme in its basic sense (e.g., treasure, Figure 1A), one its near-antonym (e.g., rubbish, Figure 1B), and one a distractor in the form of an unrelated object (e.g., beetle, Figure 1C). When shown each picture, children heard one or two sentences at a time, which they were expected to complete with the illustrated word (e.g., *What can the pirates see at the bottom of the sea? Look, there is some* …).

After seeing the first three pictures, children then saw the fourth picture (Figure 1D), and they heard a more complex story which elicited the same word that the children had produced in one of the previous slides, but this time in its abstract sense (e.g., *My friend says: Let us go searching for gold and diamonds hidden on faraway islands. I say: I do not need to go searching for that. For me, my family is the greatest* …). Eliciting the concrete sense of the word before its abstract sense was expected to optimize the chances of children producing metaphors in our study as priming has been shown to support metaphor production in young children (Gottfried, 1997).

The choice of near-antonyms and distractors was dictated by the morphosyntactic properties of the Polish polysemes. For example, the Polish polyseme *skarb* ‘treasure’ is masculine, and this required using both a Polish masculine near-antonym (*śmieć* ‘a piece of rubbish’) and a Polish masculine distractor (*żuk* ‘beetle). If we had not selected Polish words of the same gender within each four-picture block, those children who might have figured out that the fourth picture illustrated a different use (e.g., *skarb* ‘treasure’) of one of the three previously elicited words (e.g., *skarb*, i.e., gold and diamonds) may have been led to the correct answer by choosing the word that fitted the context merely in terms of gender. However, as the three words had the same gender, the children who may have figured out our test design had to choose the word that fitted the context in terms of its meaning. A distractor was introduced in the form of an unrelated object to minimize bias and the possibility that a child might re-use the correct concrete word in the metaphor condition by chance (Olofson et al., 2014).

To avoid marked facilitation from the task structure, the task was designed in such a way that there were no metaphors in the instructions, and no metaphors in the stories. This was to ensure that children’s metaphor use at the end of each target sentence was not primed by any abstract elements of the task. The order of the pictures on the screen was further randomized so that the key word in its basic sense would sometimes be presented first, and sometimes second, or third, in each four-picture block. Also, the order of all four-picture blocks was randomized to avoid floor and ceiling effects for specific types of metaphors. In total, there were four versions of the experiment available: two versions of a task which contained both English primary and Polish perceptual metaphors, and two versions of a task which contained both Polish primary and English perceptual metaphors. Each of the two versions introduced the pictures in a different order to minimize order effects.

To minimize the risk of the test design becoming transparent, especially to the older participants, and then being treated as a multiple-choice rather than metaphor production test, each four-picture block (e.g., 1A, 1B, 1C, 1D) was split up, and the fourth picture (i.e., 1D) was only introduced after the first three pictures of the following block. Table 5 illustrates how this procedure was applied to each metaphor group (primary, perceptual). This required introducing a dummy picture after the first three pictures of the first block. As two metaphor groups were included in the study (primary, perceptual), two dummy pictures were included in each version of the experiment (Polish–English and English–Polish).

### 2.4. The testing procedure

Children aged three to four took part in three half-hour sessions, either in their school, or at home. In the first session, they did RCPM, PTRJ-R and PTRJ-P; in the second, they did BPVS and ETC-3, and in the third, the metaphor elicitation task. Children aged 4–6 took part in two 45-min sessions. In the first session, they did RCPM, PTRJ-R, PTRJ-P and BPVS. In the second session, they did ETC-3 and the metaphor elicitation task. Breaks were given to children as and when required.

In the metaphor task, children were randomly allocated to one of two task-pairs: 32 children did the first, and 30 the second version of the test (Polish primary and English perceptual, or English primary and Polish perceptual). Completing the same task in both languages would have invalidated the results in the second language tested, most likely leading to better responses; alternatively, allowing a long time between testing sessions would most likely have resulted in significant drop-out rates. If the child had not produced any response after seeing the picture and hearing the narration, the picture remained on the screen and the narration was replayed one more time. A correctly used metaphor was scored as 1; any other response was scored as 0. When the child produced a novel metaphor (e.g., *behind* three o’clock instead of *after* three o’clock), which only happened with the primary metaphors of time, their novel use was counted as a correct use of the target metaphor.

### 2.5. Analyses

All the participants in the adult control group showed a performance rate of 100% on the English and Polish metaphor stories, which meant that the materials were well-designed, and the task was fit for purpose. The adult data were excluded from further analysis.

Children’s data were analyzed through an omnibus univariate ANOVA, with one dependent variable (performance), three co-variates (chronological age in months, non-verbal reasoning, and verbal abilities) and two fixed factors (metaphor type and testing language). Using continuous chronological age in months rather than age groups (i.e., three, four, five, six) was facilitated by our recruitment procedure (Section 2.1) to avoid both masking fairly large age discrepancies between children from the same age group (e.g., aged 4; 01 and 4; 11) and drawing somewhat artificial boundaries between children of fairly comparable ages (e.g., aged 3;11 and 4;00).

To allow the analysis of children’s performance in light of their lexical skills in a given language, an overall factor score (i.e., using principal component analysis) was computed for each child, indicative of both their comprehension and production abilities in each language separately. Having a language-specific factor score for each language allowed us to overcome the difficulty of comparing children’s performance in lexical tests that have different scales in English and Polish.

The data were then analyzed qualitatively. Children’s scores for each linguistic metaphor were added up per each language and children’s age to demonstrate which metaphors children performed well on at each age, and whether this pattern was the same both for both Polish and English. In the qualitative analyses, we chose to use age groups instead as the preferred means of visualization.

## 3. Results

### 3.1. Quantitative analyses

To prepare for the omnibus ANOVA, we did a Pearson one-tailed correlation test to examine the relationship between chronological age in months, non-verbal reasoning, and the verbal skills in English and in Polish. Chronological age was particularly highly correlated with non-verbal reasoning (*R* = 0.862, *p* < 0.001; Table 6), so we chose to include non-verbal reasoning only for main effects and to exclude it from interactions with any other variables tested in the final model.

**Table 6:** Correlations between age, non-verbal reasoning and verbal skills.

		Age in months	Non-verbal reasoning	Polish verbal skills	English verbal skills
Age in months	Pearson Correlation	1	0.862^**^	0.627^**^	0.818^**^
	Sig. (1-tailed)		<0.001	<0.001	<0.001
	*N*	124	124	124	124
Non-verbal reasoning	Pearson Correlation	0.862^**^	1	0.678^**^	0.733^**^
	Sig. (1-tailed)	<0.001		<0.001	<0.001
	*N*	124	124	124	124
Polish verbal skills	Pearson Correlation	0.627^**^	0.678^**^	1	0.580^**^
	Sig. (1-tailed)	<0.001	<0.001		<0.001
	*N*	124	124	124	124
English verbal skills	Pearson Correlation	0.818^**^	0.0733^**^	0.580^**^	1
	Sig. (1-tailed)	<0.001	<0.001	<0.001	
	*N*	124	124	124	124

** Correlation is significant at the 0.01 level (1-tailed).

In total, only one omnibus ANOVA was performed, using the following model: (a) main effects were included for all the variables, and (b) two-way and three-way interactions were examined between chronological age in months, verbal abilities in English and in Polish, metaphor type, and testing language, but only where our research question predicted a potential effect (Table 7).

**Table 7:** One-way ANOVA (dependent variable: performance in the metaphor task).

Source	Type III Sum of Squares	*df*	Mean square	*F*	Significance
Corrected model	479.323^a^	16	29.958	19.990	<0.001
Intercept	0.589	1	0.589	0.393	0.532
Age in months	12.839	1	12.839	8.567	0.004
Non-verbal reasoning	0.009	1	0.009	0.006	0.939
English verbal skills	18.289	1	18.289	12.204	<0.001
Polish verbal skills	8.078	1	8.078	5.390	0.022
Metaphor type	0.751	1	0.751	0.501	0.481
Testing language	2.807	1	2.807	1.873	0.174
Metaphor type/age in months	2.480	1	2.480	1.655	0.201
Metaphor type/testing language	12.741	1	12.741	8.501	0.004
Metaphor type/testing language/age in months	16.993	2	8.497	5.670	0.005
Metaphor type/testing language/English verbal skills	36.008	3	12.003	8.009	<0.001
Metaphor type/testing language/Polish verbal skills	26.008	3	8.669	5.785	0.001
Error	160.354	107	1.499		
Total	1594.000	124			
Corrected total	639.677	123			

a *R* Squared = 0.749 (Adjusted *R* squared = 0.712).

**Figure 2:**
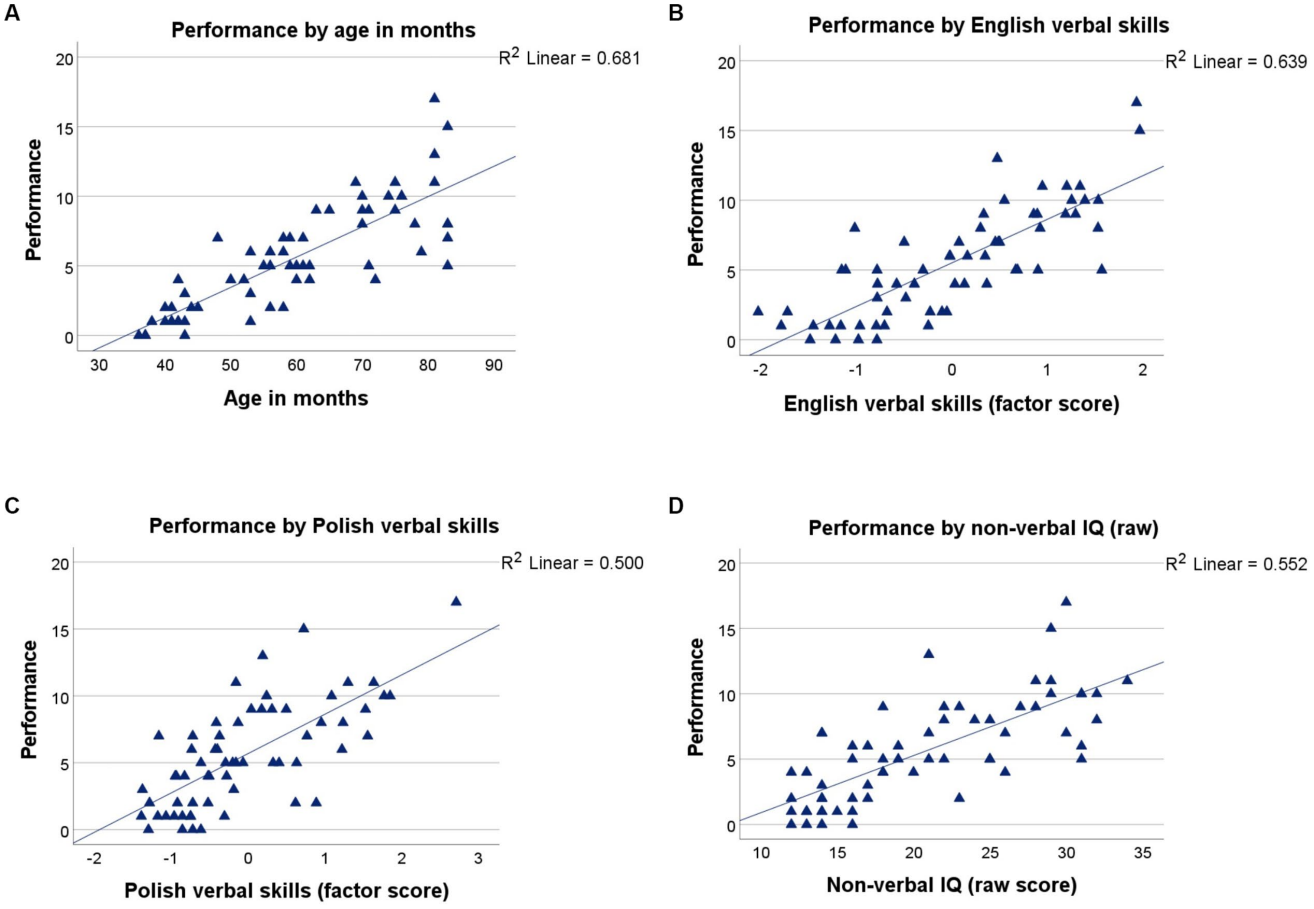
**(A)** Performance on all metaphors, by age in months (top left), **(B)** performance, by verbal skills in English (top right), **(C)** performance, by verbal skills in Polish (bottom left), and **(D)** performance, by non-verbal IQ (bottom right).

The omnibus ANOVA first considered the role of the underlying variables (chronological age, non-verbal IQ, and verbal abilities) in metaphor production in the target group. A significant main effect was found for chronological age (measured in months), *F_(1, 107)_ = 8.567, p = 0.004, η_p_^2^ = 12.839* (Figure 2A), as well as for verbal skills in English, *F_(1, 107)_ = 12.204, p < 0.001, η_p_^2^ = 18.289* (Figure 2B), and in Polish, *F_(1, 107)_ = 5.390, p = 0.022, η_p_^2^ = 8.078* (Figure 2C), but not for children’s non-verbal reasoning skills, *F_(1, 107)_ = 0.006, p = 0.939, η_p_^2^ = 0.009* (Figure 2D).

**Figure 3:**
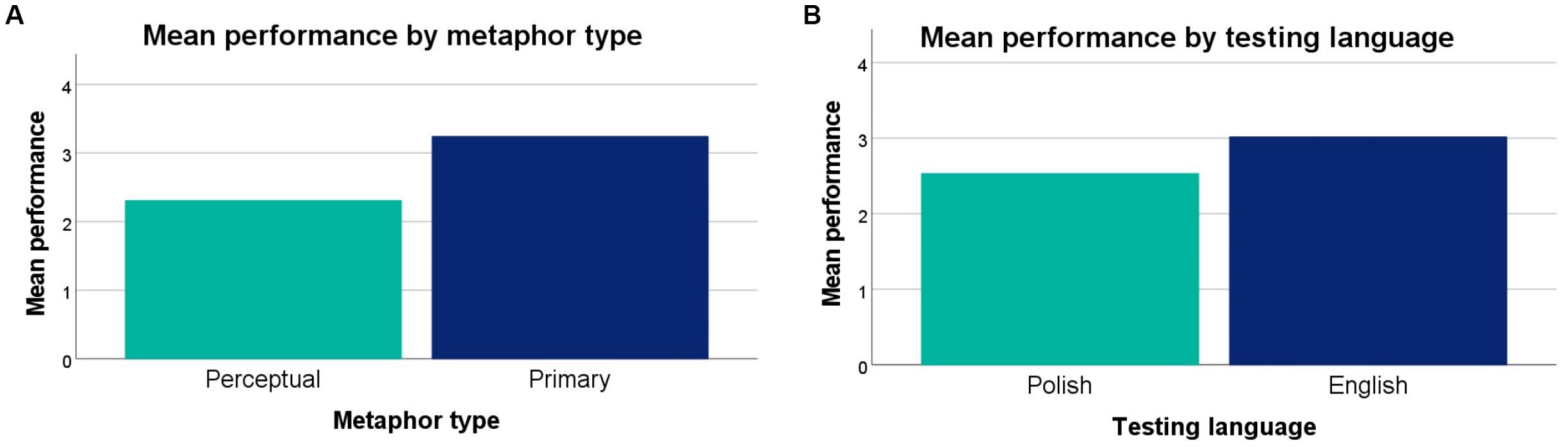
**(A)** Performance on all metaphors, by metaphor type (left) and **(B)** performance, by testing language (right).

The second set of factors to consider in metaphor production in young bilinguals were two external variables, metaphor type and language. The omnibus ANOVA revealed no significant main effect for either metaphor type, *F_(1, 107)_ = 0.501, p = 0.481, η_p_^2^ = 0.751*, or language *F_(1, 107)_ = 1.873, p = 0.174, η_p_^2^ = 2.807*, although, overall, children produced more primary than perceptual metaphors (Figure 3A), and more metaphors in English, the society language, than in Polish, their heritage language (Figure 3B). The two variables (metaphor type, testing language) were still included in the tests of interactions to determine, for example, whether the difference between metaphor type might be more significant in one language.

**Figure 4:**
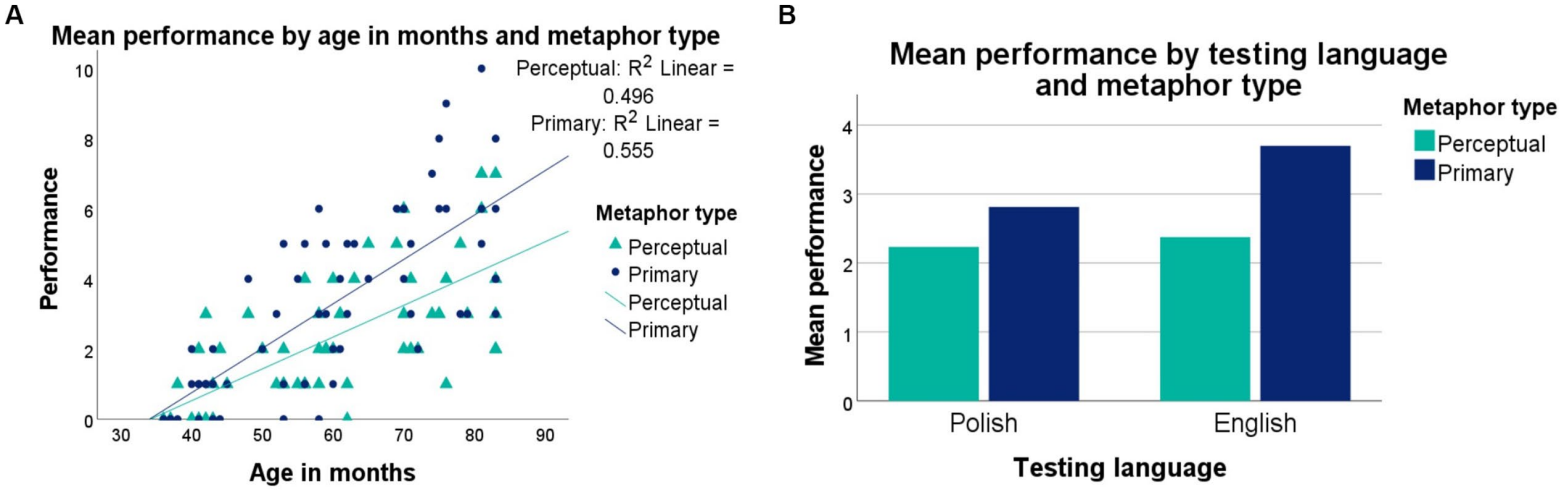
**(A)** Performance, by metaphor type and language (left), and **(B)** performance, by metaphor type and age (right).

The analysis then moved on to testing the interactions between metaphor type and chronological age, and between metaphor type and testing language. The omnibus ANOVA revealed no significant interaction between metaphor type and the children’s chronological age, *F_(1, 107)_ = 1.655, p = 0.201, η_p_^2^ = 2.480*, which suggests that the production of both primary and perceptual metaphors improves at a similar rate (Figure 4A). A significant interaction was found between metaphor type and testing language, *F_(1, 107)_ = 8.501, p = 0.004, η_p_^2^ = 12.741*. According to Figure 4B, this interaction seems to have been driven by a difference in the production of Polish and English primary rather perceptual metaphors.

**Figure 5:**
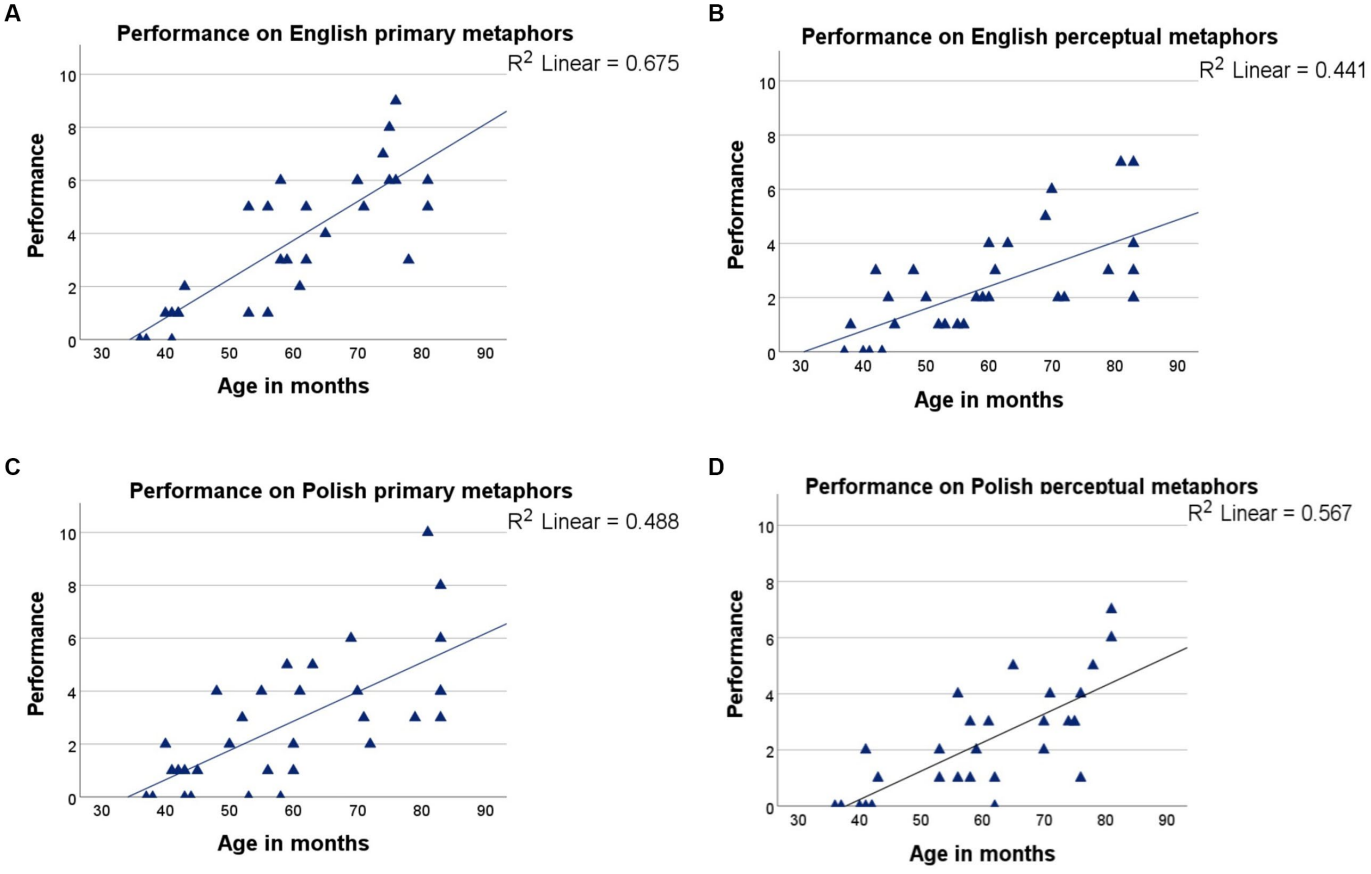
Performance by age: **(A)** English primary metaphors (top left), **(B)** English perceptual metaphors (top right), **(C)** Polish primary metaphors (bottom left), and **(D)** Polish perceptual metaphors (bottom right).

The production of primary and perceptual metaphors was then examined in Polish and English as a function of increasing chronological age. The ANOVA revealed a significant interaction between metaphor type, testing language, and children’s chronological age, *F_(2, 107)_ = 5.670, p = 0.005, η_p_^2^ = 8.497.* Chronological age could account well for the production of English primary metaphors (*R^2^ = 0.675*; Figure 5A), and quite well for that of Polish perceptual (*R^2^ = 0.567*; Figure 5D), Polish primary (*R^2^ = 0.488*; Figure 5C) and English perceptual metaphors (*R^2^ = 0.441*; Figure 5B). Overall, somewhat more children displayed performance at floor level on Polish than on English metaphors.

**Figure 6:**
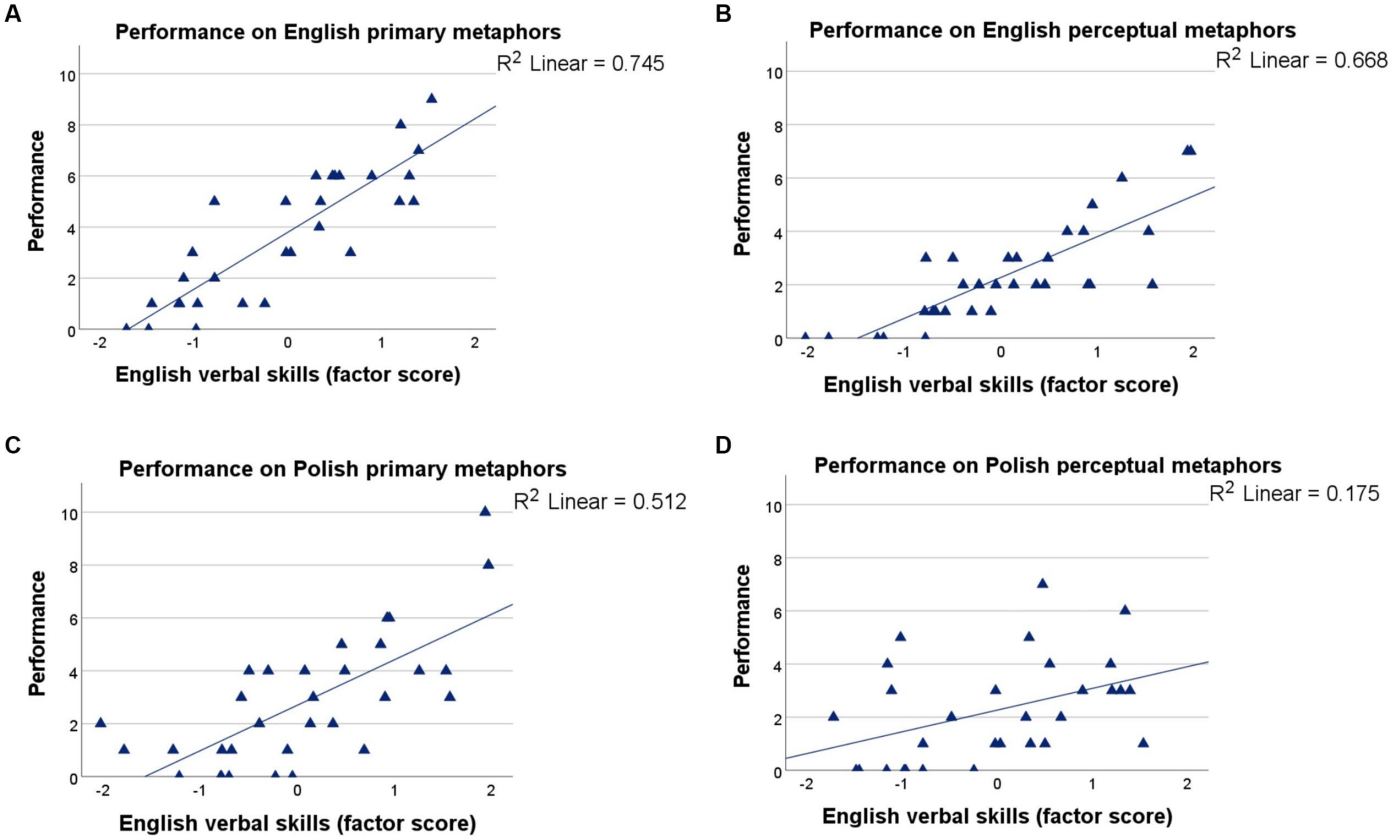
Performance by English verbal skills: **(A)** English primary metaphors (top left), **(B)** English perceptual metaphors (top right), **(C)** Polish primary metaphors (bottom left), and **(D)** Polish perceptual metaphors (bottom right).

Last, the production of Polish and English primary and perceptual metaphors was examined as a function of children’s verbal skills in English and in Polish. The omnibus ANOVA revealed a significant interaction between metaphor type, testing language, and English verbal skills, *F_(3, 107)_ = 8.009, p < 0.001, η_p_^2^ = 12.003*. According to Figure 6, English verbal skills could account well for the production of English primary (*R^2^ = 0.745*; Figure 6A) and English perceptual metaphors (*R^2^ = 0.668*; Figure 6B) and, interestingly, also for the production of Polish primary metaphors (*R^2^ = 0.512*; Figure 6C). However, they could not account well for that of Polish perceptual metaphors (*R^2^ = 0.175*; Figure 6D), showing close to no linear trend.

**Figure 7:**
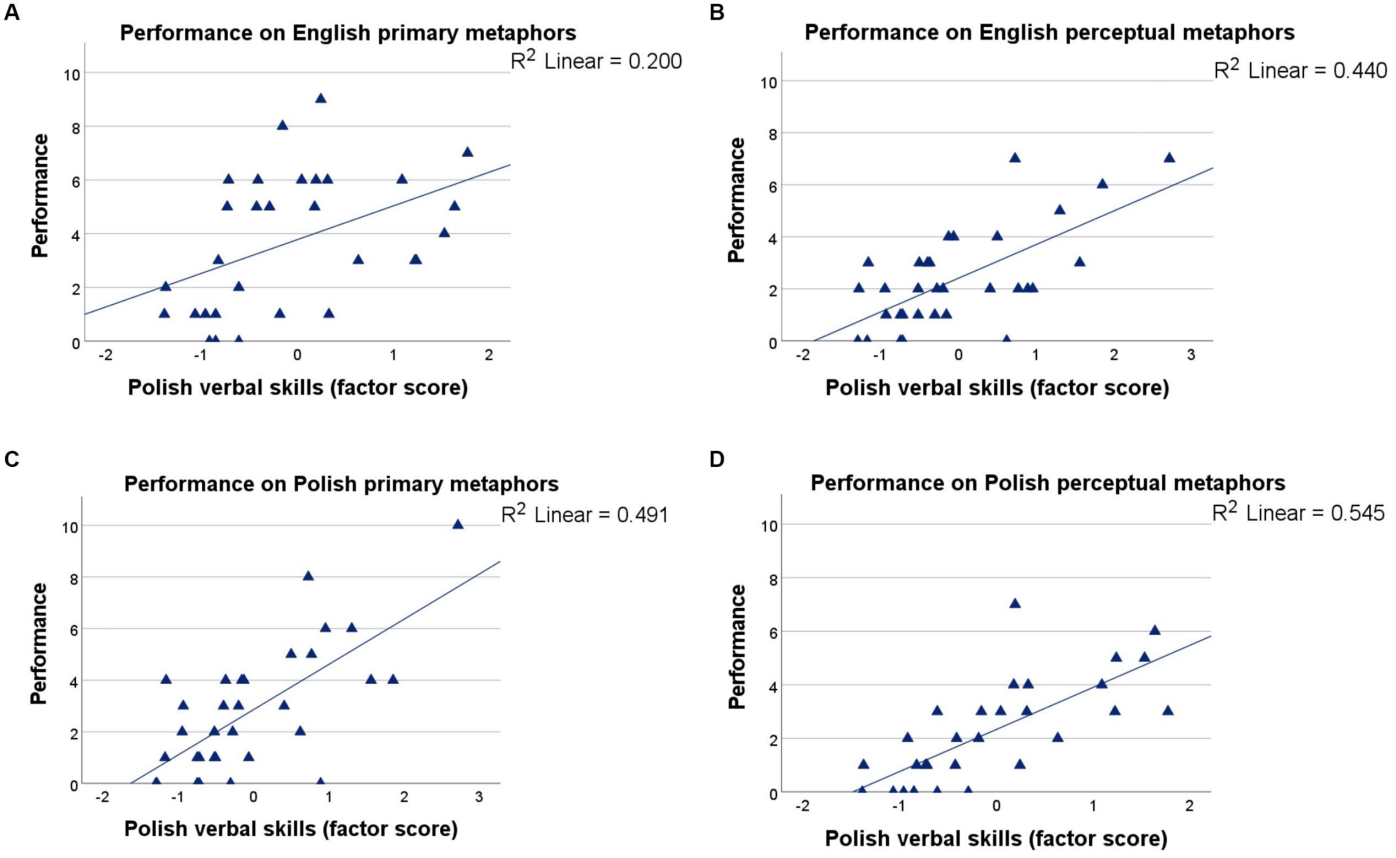
Performance by Polish verbal skills: **(A)** English primary metaphors (top left), **(B)** English perceptual metaphors (top right), **(C)** Polish primary metaphors (bottom left), and **(D)** Polish perceptual metaphors (bottom right).

There was also a significant interaction between metaphor type, testing language, and Polish verbal skills, *F_(3, 107)_ = 5.785, p = 0.001, η_p_^2^ = 8.669*. As shown in Figure 7, Polish verbal skills could account fairly well for the production of Polish perceptual (*R^2^ = 0.545*; Figure 7D) and Polish primary metaphors (*R^2^ = 0.491*; Figure 7C), somewhat less well for the production of English perceptual metaphors (*R*^2^ = 0.440; Figure 7B), with that of English primary metaphors (*R*^2^ = 0.200; Figure 7A) displaying close to no linear trend.

### 3.2. Qualitative analyses

Qualitative analyses focused on individual items. The summary of children’s scores for primary metaphors is presented in Appendix 1, and for perceptual metaphors in Appendix 2. Overall, these results demonstrate that every metaphor used in our experiment was produced by children in our study at least once. They also demonstrate that primary metaphors were accessible to children at comparable developmental stages across the two languages: the vast majority of primary metaphors displayed a similar onset of production and were produced in comparable proportions in English and in Polish. Some of the primary metaphors were available to children at a young age (e.g., You never *listen*, My stomach is about to *burst*), while others were only produced by the older participants (e.g., I have it all *under* control, You’re such an early *bird*). By contrast, there was more variation in children’s use of perceptual metaphors across Polish and English.

## 4. Discussion

The current study investigated metaphor production in 62 young bilingual speakers of English as their society language and Polish as their heritage language. As the first study in metaphor production in young bilingual children, it tested a range of key factors that impact metaphor use in the target group. The impact of these factors and their interactions will be discussed in the context of Structure Mapping Theory (SMT) and Conceptual Metaphor Theory (CMT). Usage-Based Theory (UBT) will be brought in as a novel account which can help to explain lexical effects in metaphor production.

First, we demonstrate that in a task which requires completing a story with a metaphorical word, bilingual three-year-olds can produce some conventional metaphors that are age appropriate, and that this ability improves the older they get, just like their monolingual peers (Gottfried, 1997). Of course, the extent to which children have mastered all the aspects of metaphor use at such a young age remains an open question. We did not test, for example, if children who used perceptual metaphors (e.g., *treasure*) had made a link between the word’s concrete (i.e., a chest filled with coins) and abstract meanings (i.e., a person dear to us), and whether they were fully aware of the properties transferred from the source to the target concept, or domain. However, producing a conventional metaphor may not always require such explicit knowledge; even adults use some conventional metaphors (e.g., when calling their child *pumpkin* or *pickle*) without being fully aware of the link between the source and the target and without running the risk of being perceived as non-metaphorical. At this stage, we have focused on showing that when presented with a suggestive communicative context, children can access and retrieve a word which is considered metaphorical in that specific context. Meanwhile, our youngest research participants may have struggled due to perseveration effects from the literal condition (e.g., Blakey et al., 2015): children aged four develop the ability to reliably switch to the new rule, but children aged three still find the switch challenging. In our study, children were expected to alternate between the production of literal and abstract words, which may have been challenging to the youngest group, and this remains a limitation of our study. However, as some three-year-olds did cope with the requirements of our task, these findings complement and reaffirm a newly emerging stance that metaphor use starts to emerge before children enter their primary education (Almohammadi et al., n.d.; DiPaola et al., 2019; Pouscoulous and Tomasello, 2019).

Second, we show that verbal skills do have a significant main effect on metaphor production. This result reflects some selected findings from metaphor comprehension in monolingual children (e.g., Van Herwegen et al., 2013), as well as metaphor comprehension and production in adults (e.g., Chiappe and Chiappe, 2007). We propose that in our study the significant effect of verbal skills becomes apparent due to two factors inherent to our experiment design: the use of conventional rather than novel metaphors, and a production rather than comprehension task. We suspect that unlike novel metaphors, which require an online creation of new conceptual links (Wilson and Sperber, 2012), conventional metaphors of both types are lexicalized: they are encoded in familiar words, which consolidate their linguistic nature. We also believe that unlike metaphor comprehension, metaphor production requires much greater linguistic contribution. From studies in word use, we know, for example, that even shallow and incomplete storage of an existing word form might be ‘good enough’ to allow access to its meaning (Huttenlocher, 1974), while word production requires the retrieval of information that may simply not be needed in similar levels of phonological detail in comprehension (Bock and Griffin, 2000). While metaphor comprehension tasks target primarily the child’s grasp of metaphor meaning, metaphor production tasks test a more holistic grasp of the word use, with meaning put on a par with other aspects of lexical knowledge. This may explain why we capture a significant impact of verbal skills on metaphor production, while a similar effect may not always manifest in studies in metaphor comprehension (see, e.g., DiPaola et al., 2019; Pouscoulous and Tomasello, 2019).

Third, we find no main effect of metaphor type on metaphor production, and no interaction between metaphor type and chronological age, showing that overall, primary, and perceptual metaphors were produced in similar numbers, and that they developed at a comparable pace with increasing age. This may have some implications for the theory, which ascribes distinct motivations to the way in which the two metaphor types are acquired (e.g., Grady, 2005). Studies conducted in the context of SMT see perceptual metaphor use as developing gradually alongside growing verbal skills, verbal analogy, and alternative naming (e.g., Van Herwegen et al., 2013; DiPaola et al., 2019; Pouscoulous and Tomasello, 2019); those conducted in the context of CMT see primary metaphors as triggered by the underlying mappings of the conceptual system which enable sudden growth in primary metaphor use (e.g., Özçalişkan, 2005). While our previous work has shown that primary and perceptual metaphors do differ in terms of comprehension (Almohammadi et al., n.d.) and spontaneous production (Gaskins et al., 2023), the elicitation task used in this experimental study puts them on a par with each other. This may be due to the fact that both metaphor types were embedded in stories of equal length and highly controlled (i.e., equally salient) story contexts, which posed (unusually) equal demands on the participants. It may also be due to the fact that all metaphors were tested via the use of actual words (i.e., vessels of holistic word knowledge), which might have obscured their conceptual motivations. Overall, our data show, however, that when elicited in use, primary metaphors seem to act like lexicalized perceptual metaphors (e.g., Murphy, 1996; Keysar et al., 2000; Glucksberg, 2001; Jackendoff, 2002; McGlone, 2007). This would suggest that their acquisition is governed, at least in part, by processes underlying that of any other lexical items, i.e., the accrual of activation each time a metaphor is encountered (Bybee, 2010; Schmid, 2020), and the system-wide changes in the organization of words (and metaphors) commensurate with the size or density of the lexicon in which such words (and metaphors) are embedded (Gershkoff-Stowe and Hahn, 2007). The main premise of this highly dynamic and interactive system is that the stronger the activation of a given metaphor, whether primary or perceptual, the greater its accessibility.

At the same time, our qualitative analysis of metaphors produced in each age group reveals more consistency in children’s use of primary metaphors across the two languages. It is thus possible that parity between primary and perceptual metaphors that emerges from the quantitative results may be skewed by the scores for individual linguistic metaphors. Under CMT, such cross-linguistic consistency can be explained by means of mappings that motivate the use of primary metaphors: once a mapping has been triggered, children can use primary metaphors related to that mapping to similar degrees in their two languages. Meanwhile, less consistent use of perceptual metaphors in English and in Polish can be explained within the context of SMT: perceptual metaphors are linguistically, contextually, and culturally sensitive so their use is naturally more varied across speakers. Bilingual acquisition experiences particularly high levels of variation, driven by the typically uneven levels of exposure to the two languages (e.g., David and Li, 2008; Hoff et al., 2012), and the different numbers of speakers children interact with in each language (e.g., De Houwer, 2007; Jia and Fuse 2007; Quay and Montanari 2016; Unsworth, 2017). Apart from the quantitative factors that impact the constellation of their input, bilingual children are also likely to have very different qualitative experience of their two languages from different levels of engagement with literature, television, stories, to mention but a few factors (Unsworth, 2017). All this means that they will all have very different opportunities to encounter and acquire perceptual metaphors.

While we find no significant main effect for metaphor type, there is a significant interaction between metaphor type and testing language, driven by an apparent difference between the two languages in the production of primary metaphors. This observation is inconsistent with CMT, which predicts that primary metaphors are embodiment driven, and should emerge on a similar schedule between the two languages (e.g., Özçalişkan, 2005); it also challenges SMT, which assumes that perceptual metaphors are language sensitive and should emerge on different schedules across languages (e.g., Gentner et al., 2001).

Perceptual metaphors used in our experiment were encoded in nouns and verbs, and the primary metaphors in verbs, adverbs, adjectives, and prepositions, which represent radically different usage frequencies, though it is important to remember that all items were matched for familiarity. Children tend to know many types of nouns but use each of them with low frequency counts, while they have smaller pools of verbs, adverbs, adjectives, and closed-class items but use them more frequently in speech (Goodman et al., 2008). Compare, for example, word usage frequencies in the speech of a child called Sadie with English as her main language and Polish as her heritage language (Gaskins, 2018): when video recorded for 15 h around the age of two and a half, Sadie used English nouns and adjectives on average less frequently (*M* = 4.49; *M* = 3.75) than verbs (*M* = 6.70), adverbs (*M* = 16.05), or closed-class items (*M* = 35.12). Fewer opportunities to use Polish than English words translated into lower usage frequencies, as in the same recordings, Sadie showed a similar average use of Polish nouns (*M* = 4.16), and verbs (*M* = 7.20), but a lower average use of Polish adjectives (0), adverbs (*M* = 4.25) and closed-class items (*M* = 3.58; Gaskins, 2018). As the primary metaphors used in our experiment with bilingual speakers of English and Polish were encoded in words that tend to occur with higher frequencies, this alone might explain why children produce more primary than perceptual metaphors, and why primary metaphors in the society language are produced in higher numbers than those used in the heritage language. UBT may also, to an extent, explain similar numbers of perceptual metaphors produced in English and in Polish. As nouns tend to be recycled to a similar extent (Goodman et al., 2008; Gaskins, 2018), they are likely to display similar usage frequencies across the two languages than other parts of speech.

Even though we did not capture an interaction between the children’s chronological age and metaphor type, our study reveals a significant interaction between chronological age, metaphor type, and testing language. Chronological age appears to account well for the ability to produce all four types of metaphors, but it seems to have the most significant effect on English primary metaphors. This observation is not inconsistent with CMT; as children become more aware of their environment and how it works, their increasing age and their growing knowledge of the world should have the most impact on their use of primary metaphors in their stronger language. This observation can also be explained in the context of UBT; as most of the tested children are dominant in English and have larger lexicons in English than in Polish, they require the least activation to produce primary metaphors which are, in theory, the most established in the neural network through frequency-based accrual of activation. In a sense, primary metaphors, often encoded in function rather than content words, are like some aspects of grammar; once the child starts to produce them, they require little input and experience a healthy growth, especially in the stronger language, which is frequently reinforced by the society and education (Unsworth, 2014). Meanwhile, primary metaphors in the heritage language, and perceptual metaphors in both languages which are typically encoded in nouns, experience a less stable development.

Following this, we found a significant interaction between metaphor type, testing language, and English verbal skills, driven by both types of English metaphors and Polish primary metaphors, but not Polish perceptual metaphors. This observation is also consistent with UBT. As most of the tested children hear more English than Polish on a daily basis, and have larger lexicons in English than in Polish, lexical networks in their English lexicons are likely to be more interconnected than those in Polish. The higher their English verbal skills, the easier it is for them to access and retrieve English metaphors, especially primary metaphors encoded in high frequency words which are embedded in dense lexical networks. Good verbal skills in English also have a significant impact on the production of Polish primary metaphors, as they seem to reinforce their use in the lesser practiced language. This probably happens in two ways. Our first explanation, which plays into the hands of UBT, could be that the two lexical systems are abstracted via skills of semantic categorization, which may partly contribute to developing a shared conceptual representation between English and Polish primary metaphors. The stronger the English skills in one specific area, the sooner abstract categories are formed and this, in turn, supports the production of English as well as Polish words. For example, when accessing the metaphor *Jej dzień był bardzo **długi*** ‘Her day was very long’, children are likely to rely on their experience with English when producing the equivalent metaphor in Polish and vice versa. Under CMT, on the other hand, the interaction with English verbal skills could be explained by a shared underlying mapping which drives the selection of language-, context-, and speaker-specific vocabulary; when a language-appropriate match is not found, an equivalent in the other language may be selected. Growing verbal skills in English also have a significant effect on the production of English but not Polish perceptual metaphors. It is possible that due to low usage frequencies of nouns, there is a limited scope for English perceptual metaphors to achieve any abstraction and exert an influence on their lesser used Polish equivalents.

There is also a significant interaction between metaphor type, testing language, and Polish verbal skills, which seems to be driven by Polish primary and perceptual metaphors as well as English perceptual but not English primary metaphors. The effects of Polish verbal skills on Polish metaphors are a given in the context of frequency-based activation (UBT); the effects of Polish verbal skills on English perceptual metaphors can be explained with reference to categorization (SMT). Under SMT, perceptual metaphors are treated as a linguistic phenomenon, which is sensitive to verbal skills and semantic categorization. In the context of bilingualism, the emerging semantic categories are shaped by input from two languages (English and Polish), which explains why Polish verbal skills would also affect the use of perceptual metaphors in English. However, the role of semantic categorization in bilingual use of perceptual metaphors, or any metaphors for that matter, is only our speculation and it requires more specific testing in the future.

One limitation of our study may be the fact that it tested metaphor use across different children instead of focusing on one group and testing them repeatedly as they grew older. Likewise, children were tested on each metaphor type only in one, not two, languages. This type of design, as well as the fact that it used a potentially varied bilingual cohort, could have obscured some factors that contribute to metaphor production. To address these issues, future research should consider following one group of children longitudinally over a period of several years. Another limitation of our study is its focus on a restricted number of factors driving metaphor production. Future studies should explore additional constructs, such as verbal analogy (e.g., Pouscoulous and Tomasello, 2019), as well as executive functions (e.g., working memory), previously linked to individual differences in metaphor comprehension in adults (Chiappe and Chiappe, 2007; Columbus et al., 2015). Last but not least, to provide a more representative picture of bilingual metaphor acquisition, studies should compare the use of metaphors that overlap between the two languages with those which do not.

## 5. Concluding remarks

Metaphor acquisition frameworks have emerged mostly from research in metaphor comprehension in monolingual children, with the onset of metaphor production largely undocumented, and unexplained. Our study in metaphor production demonstrates that testing production skills under experimental conditions is possible, as long as the use of metaphors is neatly scaffolded by a suggestive context whose simplicity reflects the state of children’s world knowledge, and as long as prompts are short and straightforward for children of that age, read out in an expressive and engaging manner, and illustrated with age-appropriate pictures. Focus on bilingual metaphor production helps us to disentangle verbal and non-verbal skills in metaphor production. As we focus on conventional rather than novel metaphors, and production rather than comprehension, we capture strong effects of verbal skills in both languages on metaphor use. Our results bridge a gap between two contrasting accounts of metaphor acquisition and highlight usage-based effects in their acquisition. At the same time, they show that metaphor acquisition cannot be explained by drawing on only one theoretical account.

## Data availability statement

The original contributions presented in the study are included in the article/supplementary material, further inquiries can be directed to the corresponding author.

## Ethics statement

The studies involving human participants were reviewed and approved by King’s Research Office Committee, King’s College London. Written informed consent to participate in this study was provided by the participants’ legal guardian/next of kin.

## Author contributions

DG and GR designed the study, analyzed the data, and revised the manuscript. DG collected the data and wrote the first draft of the manuscript. Both authors contributed to the article and approved the submitted version.

## Funding

This project is funded by grant ECF-2020-229 from the Leverhulme Trust awarded to DG.

## Conflict of interest

The authors declare that the research was conducted in the absence of any commercial or financial relationships that could be construed as a potential conflict of interest.

## Publisher’s note

All claims expressed in this article are solely those of the authors and do not necessarily represent those of their affiliated organizations, or those of the publisher, the editors and the reviewers. Any product that may be evaluated in this article, or claim that may be made by its manufacturer, is not guaranteed or endorsed by the publisher.

## Appendix 1

The number of primary metaphors elicited from children.

**Table:**

Primary metaphor	Age 3–4	Age 4–5	Age 5–6	Age 6–7
*Her day was very **long**.*	0	3	5	4
*Jej dzień był bardzo **długi**.*	0	4	3	6
*Number 4 is **after** number 3.*	1	3	7	7
*Numer 4 jest **po** numerze 3.*	0	2	4	6
*Because you never **listen**.*	3	5	5	8
*Bo się nigdy nie **słuchasz**.*	3	5	5	8
*(…) when winter **comes**.*	1	3	6	8
*(…) jak zima **przyjdzie**.*	1	4	5	6
*Thank you. You’re so **sweet**.*	1	3	3	5
*Dziękuję. Jesteś bardzo **słodki**.*	1	1	3	3
*I have it all **under** control.*	0	1	1	3
*Mam tu wszystko **pod** kontrolą.*	0	0	1	3
*How is it **going**?*	0	1	1	4
*Jak ci tam **idzie**?*	0	2	3	4
*The price was too **high**.*	0	1	2	4
*Cena była za **wysoka**.*	0	0	0	2
*We are very **close**.*	0	1	0	5
*Jesteśmy ze sobą bardzo **blisko**.*	0	1	2	2
*(…) a promise you cannot **break**.*	0	3	1	2
*(…) słowa nie możesz **złamać**.*	0	0	0	1

## Appendix 2

The number of perceptual metaphors elicited from children.

**Table:**

Perceptual metaphors	Age 3–4	Age 4–5	Age 5–6	Age 6–7
*My family is the greatest **treasure**.*	0	0	2	3
*Moja rodzina to największy **skarb**.*	0	1	1	3
*You are my **sunshine**.*	1	4	1	1
*Ty jesteś moim **słoneczkiem**.*	1	1	0	2
*You’re such a busy **bee**.*	0	1	1	0
*Ale z ciebie pracowita **pszczółka**.*	0	0	0	1
*You are a real **star**.*	1	0	2	4
*Prawdziwa z ciebie **gwiazda**!*	0	1	2	3
*You’re such an early **bird**.*	0	0	0	2
*Ale z ciebie ranny **ptaszek**.*	1	0	0	3
*Lion is the **king**.*	4	3	5	7
*Lew to **król**.*	0	2	4	5
*You’re such a **pig**.*	0	0	2	3
*Ale z ciebie **świnka**.*	0	1	1	1
*Or she will not be able to **catch** it.*	1	3	3	3
*Żeby zdążyła autobus **złapać**.*	0	2	5	5
*My stomach is about to **burst**.*	0	3	5	7
*Aż brzuch mi **pęka**.*	2	6	4	7
*You can just **drop in**.*	0	0	0	1
*Możecie do nas **wpaść**.*	0	1	1	2
